# Relationship Between Serum Amino Acid Levels and Bone Mineral Density: A Mendelian Randomization Study

**DOI:** 10.3389/fendo.2021.763538

**Published:** 2021-11-09

**Authors:** Zhiyong Cui, Hui Feng, Baichuan He, Jinyao He, Yun Tian

**Affiliations:** ^1^ Department of Orthopaedics, Peking University Third Hospital, Beijing, China; ^2^ Peking University Third Hospital, Beijing Key Laboratory of Spinal Disease Research, Beijing, China; ^3^ Engineering Research Center of Bone and Joint Precision Medicine, Ministry of Education, Beijing, China

**Keywords:** amino acid, bone mineral density – BMD, Mendelian randomization, valine, tyrosine, isoleucine

## Abstract

**Background:**

This study aimed to explore the association between serum amino acids (AAs) levels and bone mineral density (BMD).

**Methods:**

We performed a two-sample Mendelian randomization (MR) analysis to analyze the associations between the levels of eight AAs and BMD values by using summary-level genome-wide association study (GWAS) data. We applied the MR Steiger filtering method and MR Pleiotropy RESidual Sum and Outlier (MR-PRESSO) global test to check for and remove single nucleotide polymorphisms (SNPs) that were horizontally pleiotropic. The associations were estimated with the inverse variance weighted (IVW), MR-Egger, weighted median and MR Robust Adjusted Profile Score (MR.RAPS) methods.

**Results:**

Our study found that genetically increased isoleucine (Ile) [IVW: effect = 0.1601, 95% confidence interval (CI) = 0.0604 ~ 0.2597, p = 0.0016] and valine (Val) levels (IVW: effect = 0.0953, 95% CI = 0.0251 ~ 0.1655, p = 0.0078) were positively associated with total body BMD (TB-BMD). The results also revealed that genetically increased tyrosine (Tyr) levels were negatively associated with TB-BMD (IVW: effect = -0.1091, 95% CI = -0.1863 ~ -0.0320, p = 0.0055).

**Conclusions:**

In this study, associations between serum AA levels and BMD were established. These findings underscore the important role that serum AAs play in the development of osteoporosis and provide evidence that osteoporosis can be prevented and treated by the intake of certain AAs.

## Introduction

Osteoporosis is the most common bone disease, and it is characterized by low bone mass, bone tissue deterioration and bone structure disruption (1). Osteoporosis is also the reason for fragility fractures, and the most common fracture sites are the spine, hip and distal forearm. The one-year estimated mortality of hip fractures in mainland China is 13.96% (2). Therefore, osteoporosis is a major threat to an enormous number of people and exacts a terrible toll on elderly adults, who constitute a rapidly growing population in the world. The measurement of bone mineral density (BMD) has been proven to be an effective method for diagnosing osteoporosis and assessing the risk of fragility fracture (3). Although osteoporosis is an important and common public health problem, the mechanisms and risk factors underlying osteoporosis and BMD are still poorly understood.

Optimal intake of certain nutrients, such as calcium and selenium, has a substantial impact on BMD and is positively correlated with BMD (4). Dietary proteins are important nutrients for maintaining musculoskeletal health. Both bone and muscle are lost with age, with up to 1% lost per year after age 50, and increased dietary protein intake with age is recommended to ameliorate this loss (5). A systematic review and meta-analysis performed in 2009 of published papers from January 1966 to July 2008 showed that protein supplementation had a significant positive influence on lumbar spine BMD in human adults; moreover, nearly all published cross-sectional studies demonstrated a positive association between dietary protein intake and bone health (6). As the main components of proteins, amino acids (AAs) also play an important role in regulating bone metabolism. However, a clear consensus has not been reached on the role of AAs in bone health because AAs may have competing effects on bone. A recent study in monozygotic twins demonstrated the genetically independent benefit of several specific AAs for bone health (7). One large-scale cohort study by Su et al. (8) suggested that a specific AA profile is correlated with greater BMD and lower subsequent fracture risk, independent of diet and lifestyle factors. Male patients with idiopathic osteoporosis also presented changes in free AA profiles, which indicated the role of AA utilization in osteoporosis (9). However, observational studies that estimated causal inference have numerous inherent limitations, such as reverse causality and confounding effects, thus making the interpretation of these associations difficult and their meaning uncertain (10).

Mendelian randomization (MR) analyses can overcome the limitations of conventional studies by using single nucleotide polymorphisms (SNPs) as instrumental variables (IVs) for assessing the causal effect of a risk factor (exposure) on an outcome (11). MR relies on three assumptions: (a) the genetic variant is associated with the exposure; (b) the genetic variant is not associated with confounders; and (c) the genetic variant influences the outcome only through the exposure. A two-sample MR obtains IV-exposure and IV-outcome associations from two different sets of participants. The IVs used in MR are derived from genome-wide association studies (GWAS) and available due to the development of high-throughput genomic technologies. Therefore, in this study, we used the MR approach to explore the causal effect of circulating AA levels on total body and site-specific BMD. This approach can provide estimates of the effects of traits while reducing bias due to confounding and reverse causation. The design strategy for the two-sample MR in our study is shown in **Figure S1** in the Supplementary Material.

## Methods

We performed a two-sample MR analyses to study the effect of AA levels on BMD values. Our approach relied upon summary-level GWAS data to obtain MR estimates (12, 13). We selected SNPs with a genome-wide association (p<5E-08), with independent inheritance (R^2^<0.001), and without linkage disequilibrium (LD) with each AA as IVs. Proxy SNPs (R^2^>0.9) from LDlink (https://ldlink.nci.nih.gov/) were used when the SNPs were not available for the outcome (14). To estimate the LD level, we selected the reference sample formed by European ancestral individuals from the 1000 Genomes Project (15). Palindromic SNPs with intermediate allele frequencies (palindromic SNPs refer to SNPs with the A/T or G/C alleles and “intermediate allele frequencies” refer to 0.01<allele frequency<0.30) were excluded from the selected instrument SNPs. We also calculated the F statistics for the SNPs to measure the strength of the instruments. IVs with an F statistic less than 10 were excluded and frequently labeled “weak instruments” (16). SNPs with a minor allele frequency (MAF) of < 0.01 were also excluded to avoid potential the statistical bias from the original GWAS since they usually carry with low confidence. Moreover, we used the PhenoScanner tool (17, 18) to check whether any of the selected SNPs were associated with potential confounders at risk of affecting BMD. We set the threshold at genome-wide significance (p<5E-08) when using the PhenoScanner tool.

The summary data for the associations between SNPs and AAs were retrieved from the Nightingale Health UK Biobank Initiative. The Finnish innovator of an internationally recognized blood biomarker technology for studying chronic diseases will analyze the biomarker profiles of 500,000 blood samples from the UK Biobank. Nightingale’s biomarker profiling technology will be used to analyze the UK Biobank blood samples by measuring metabolic biomarkers found by recent studies. The UK Biobank recruited 502,639 European participants aged 37~70 years in 22 assessment centers across the UK. All study participants reached the assessment centers by their own means, and enrollment was not performed at nursing homes. All participants provided written informed consent, and ethical approval was obtained from the North West Multicenter Research Ethics Committee. Blood samples were drawn at baseline between 2007 and 2010 (19). A random subset of nonfasting baseline plasma samples from 118,466 individuals and 1,298 replication samples were measured using high-throughput nuclear magnetic resonance spectroscopy (Nightingale Health Plc; biomarker quantification version 2020), which provided simultaneous quantification of 249 metabolic biomarkers, including AAs, routine lipids, lipoprotein subclass, fatty acid composition, and other low-molecular weight metabolites, such as ketone bodies and glycolysis metabolites quantified in molar concentration units, in a single assay (20). The metabolic biomarker dataset from the Nightingale Health UK Biobank Initiative was made available for the research community through the IEU GWAS database, which is a database of genetic associations in the GWAS summary datasets (https://gwas.mrcieu.ac.uk/) (13). We only focused on the particular set of AAs and extracted summary statistics about eight single AAs, namely, alanine (Ala), glutamine (Gln), histidine (His), isoleucine (Ile), leucine (Leu), phenylalanine (Phe), tyrosine (Tyr) and valine (Val), because we wanted to investigate whether AA metabolism could be associated with BMD values.

We extracted summary statistics on femoral neck (FN), lumbar spine (LS) and forearm (FA) BMD (g/cm^2) from the GEnetic Factors for OSteoporosis Consortium website (21). Genetic variants with large effects on BMD were identified in 53,236 individuals of European ancestry. Genetic variants with a minor allele frequency (MAF)>0.5% were tested for their effects on femoral neck, lumbar spine (L1-4), and forearm BMD, and the values were adjusted for sex, age, age^2^, weight and standardized to have a mean of zero and a standard deviation of one because different dual-energy X-ray absorptiometry (DXA) machines have known systematic differences in BMD measurements. The summary-level data for total body BMD (TB-BMD) (g/cm^2) were extracted from one GWAS meta-analyses including 30 epidemiological studies comprising individuals from populations across America, Europe, and Australia, with a variety of designs and participant characteristics (22). Most participants in the study were from population-based cohorts of European ancestry, two cohorts comprised African American individuals, and four cohorts included individuals with a mixed background. TB-BMD was also measured by DXA following standard manufacturer protocols. TB-BMD values were corrected for age, weight, height, and genomic principal components. The detailed characteristics of the GWAS associated with exposures and outcomes are shown in **Table S1** in the Supplementary Material.

We applied the MR Pleiotropy RESidual Sum and Outlier (MR-PRESSO) global test (23) to remove SNPs that were horizontal pleiotropic outliers to reduce heterogeneity in the estimate of the causal effect. We conducted this analysis by using the MR-PRESSO R package (https://github.com/rondolab/MR-PRESSO). The number of distributions was set to 1,000 and the threshold was set to 0.05. Moreover, we applied MR Steiger filtering (24) as implemented in the TwoSampleMR R package to test the causal direction of each of the extracted SNPs on the exposures and outcomes. This approach calculated the variance explained in the exposure and the outcome by the instrument SNPs and tests whether the variance in the outcome is less than the exposure. A “TRUE” MR Steiger result suggested causality in the expected direction, while a “FALSE” result suggested causality in the reverse direction. We excluded SNPs with “FALSE” results, which meant that it showed evidence of primarily affecting outcomes rather than exposures.

We conducted the MR with inverse variance weighted (IVW) (13, 25), MR-Egger (26, 27) and Weighted median estimate methods (28). The IVW method uses a meta-analysis approach to combine the Wald ratios of the causal effects of each SNP and can provide the most precise estimates (13, 25). The Weighted median estimate provides a reliable effect estimate of the causal effect when at least 50% of the weight in the analysis comes from effective IVs (28). MR-Egger regression is used to create a weighted linear regression of the outcome coefficients with the exposure coefficients. The slope of the weighted regression line provides an asymptotically unbiased causal estimate of the exposure on the outcome if the INSIDE (instrument strength is independent of direct effect) assumption is met. In addition, the intercept of the MR-Egger regression line was used to quantify the amount of horizontal pleiotropy present in the data averaged across the genetic instruments (26, 27). Under the INSIDE assumption, the MR-Egger intercept test identifies horizontal pleiotropy if the intercept from the MR-Egger analysis is not equal to zero (27). We also calculated the MR Robust Adjusted Profile Score (MR.RAPS) to estimate the causal effects because it can lead to considerably higher statistical power than conventional MR analysis (29). MR.RAPS considers the measurement error in SNP-exposure effects, and it is unbiased when weak instruments are used and robust to systematic and idiosyncratic pleiotropy (29). The MR.RAPS method can also alleviate but cannot solve the problem of horizontal pleiotropy (29). We used the IVW (30) method to detect heterogeneity, which was quantified by the Cochran Q statistic. Moreover, we also performed multivariable MR (MVMR) analysis to control for genetic associations of AAs with some BMD potential risk factors, such as alcohol consumption, BMI and education attainment to adjust for the effect of confounders. The summary-level data of alcohol consumption were extracted from the GWAS study in the UK Biobank (UKB) sample of white British individuals (31), BMI were extracted from the meta-analysis of GWAS in European adults (32) and education attainment were extracted from the GWAS conducted in a discovery sample of 101,069 individuals and a replication sample of 25,490 (33). We estimated the power of our study according to a method suggested by Brion et al. (34). This method uses a noncentrality parameter to calculate the statistical power of the continuous outcome and an approximate linear model on the observed binary scale adapted for binary outcome. The method required several parameters to estimate the power. For the continuous outcomes, the first parameter was the proportion of phenotypic variation (r2) explained by IV SNPs, which was estimated on the original GWAS. The second was the effect size of the exposure to the outcome at the epidemiological level, which was estimated from another independent observational cohort (8). Addition parameters included the sample size and standard deviation (SD) of exposure and outcome. The summary-level MR analysis was performed by the TwoSampleMR package (version 0.5.0) in R (version 3.6.1, the R foundation). The statistical tests of the MR analysis were two-sided, and the results of the MR analyses regarding the causal effects of AAs on BMD were considered statistically significant at a Bonferroni-corrected p<0.0125 (e.g., 0.050/4 outcomes). Relationships for which the p value was below 0.05 but above 0.0125 were considered nominally significant.

## Results

According to the SNP selection criteria, we first extracted 36, 46, 17, 10, 17, 9, 38 and 22 significant genome-wide and independently inherited SNPs associated with eight AAs. When extracting the corresponding SNPs for outcomes, we had to exclude some SNPs that were absent and no proxy SNPs in high LD (R^2^>0.9) found from LDlink in the summary statistics of outcomes. We also removed the palindromic SNPs when harmonizing the effect of IVs and excluded the SNPs with false causal direction identified by the MR Steiger filtering. Moreover, when using the PhenoScanner tool, we excluded some SNPs that were associated with confounders, which were proved to be causally associated with BMD such as body mass index (BMI), weight, calcium and low density lipoprotein (LDL) (4, 35), which might violate the second assumption of MR. We also excluded the horizontal pleiotropic outliers through the MR-PRESSO global test. The selection process and the reasons for selecting the SNPs are described in detail in **Figures S2–9**. The final numbers of SNPs included in the MR are presented in **Tables 1**–**4**. For all the included IVs, the F statistics were above 10 (e.g., Ala: ranging from 30.0542 to 249.5155 for FN-BMD; from 30.0542 to 249.5155 for LS-BMD; from 30.8591 to 249.5155 for FA-BMD and from 30.0542 to 249.5155 for TB-BMD), which indicated that they satisfy the strong relevance assumption of MR and that weak instrument bias would not substantially influence the estimations of causal effects. We also confirmed the true causal direction for the included SNPs with the MR Steiger method. The proportion of phenotypic variation explained by each genetic variant was also calculated. The detailed characteristics of the included SNPs are shown in **Tables S2–9**.

**Table 1:** MR estimates of the causal effects of AAs on FN-BMD using various analysis methods.

Exposures	Methods	Number of SNPs	Effect	95% CI	MR p-value	Cochran Q statistic	Heterogeneity p-value	MR-Egger		MR-PRESSO
								Intercept	Intercept p-value	Global test p-value
Ala	IVW	25	0.0056	-0.0938~0.1050	0.9126	32.4024	0.1173			
	MR-Egger	25	0.1210	-0.1252~0.3672	0.3454			-0.0053	0.3257	
	Weighted median	25	0.0860	-0.0402~0.2121	0.1817					
	MR.RAPS	25	0.0124	-0.0855~0.1102	0.8045					
	MR-PRESSO	25	0.0056	-0.0938~0.1050	0.9135					0.1015
Gln	IVW	27	-0.0457	-0.1068~0.0153	0.1417	29.7737	0.2771			
	MR-Egger	27	-0.0434	-0.1290~0.0422	0.3293			-0.0002	0.9395	
	Weighted median	27	-0.0507	-0.1172~0.0158	0.1350					
	MR.RAPS	27	-0.0431	-0.1081~0.0218	0.1931					
	MR-PRESSO	27	-0.0457	-0.1068~0.0153	0.1537					0.3661
His	IVW	8	0.1150	-0.0247~0.2547	0.1067	4.7754	0.6874			
	MR-Egger	8	0.2536	-0.4301~0.9372	0.4946			-0.0094	0.6990	
	Weighted median	8	0.0494	-0.1309~0.2298	0.5910					
	MR.RAPS	8	0.1159	-0.0292~0.2609	0.1174					
	MR-PRESSO	8	0.1150	-0.0004~0.2304	0.0918					0.6517
Ile	IVW	7	0.1409	-0.0285~0.3103	0.1030	9.0653	0.1699			
	MR-Egger	7	0.2172	-0.2811~0.7154	0.4320			-0.0041	0.7601	
	Weighted median	7	0.1532	-0.0397~0.3461	0.1195					
	MR.RAPS	7	0.1734	-0.0037~0.3506	0.0550					
	MR-PRESSO	7	0.1409	-0.0285~0.3103	0.1541					0.1655
Leu	IVW	10	0.0411	-0.0900~0.1722	0.5391	10.5394	0.3086			
	MR-Egger	10	0.0454	-0.2660~0.3569	0.7821			-0.0003	0.9762	
	Weighted median	10	0.0406	-0.1056~0.1869	0.5862					
	MR.RAPS	10	0.0528	-0.0726~0.1783	0.4092					
	MR-PRESSO	10	0.0411	-0.0900~0.1722	0.5543					0.4418
Phe	IVW	7	-0.0081	-0.1285~0.1122	0.8948	8.5048	0.2034			
	MR-Egger	7	-0.0904	-0.3683~0.1875	0.5518			0.0060	0.5432	
	Weighted median	7	-0.0380	-0.1586~0.0826	0.5368					
	MR.RAPS	7	-0.0107	-0.1306~0.1093	0.8615					
	MR-PRESSO	7	-0.0081	-0.1285~0.1122	0.8991					0.3286
Tyr	IVW	25	-0.0111	-0.0867~0.0644	0.7724	26.5534	0.3257			
	MR-Egger	25	-0.0026	-0.1189~0.1138	0.9657			-0.0007	0.8490	
	Weighted median	25	-0.0119	-0.1116~0.0877	0.8143					
	MR.RAPS	25	-0.0111	-0.0871~0.0649	0.7739					
	MR-PRESSO	25	-0.0111	-0.0867~0.0644	0.7749					0.3703
Val	IVW	12	0.0719	-0.0294~0.1732	0.1640	8.2676	0.6892			
	MR-Egger	12	0.1122	-0.0689~0.2934	0.2525			-0.0028	0.6103	
	Weighted median	12	0.0393	-0.0887~0.1673	0.5469					
	MR.RAPS	12	0.0738	-0.0310~0.1785	0.1675					
	MR-PRESSO	12	0.0719	-0.0159~0.1597	0.1367					0.6874

AAs, amino acids; Ala, alanine; Gln, glutamine; His, histidine; Ile, isoleucine; Leu, leucine; Phe, phenylalanine; Tyr, tyrosine; Val, valine; BMD, bone mineral density; FN-BMD, femoral neck BMD; SNPs, single nucleotide polymorphisms; MR, Mendelian randomization; IVW, inverse variance weighted; MR.RAPS, MR Robust Adjusted Profile Score; MR-PRESSO, MR Pleiotropy RESidual Sum and Outlier; Effect, the causal effects of 1-SD increase of AAs on BMD; SD, standard deviation; CI, confidence interval.

**Table 2:** MR estimates of the causal effects of AAs on LS-BMD using various analysis methods.

Exposures	Methods	Number of SNPs	Effect	95% CI	MR p-value	Cochran Q statistic	Heterogeneity p-value	MR-Egger		MR-PRESSO
								Intercept	Intercept p-value	Global test p-value
Ala	IVW	20	-0.0517	-0.1676~0.0642	0.3822	22.0655	0.2810			
	MR-Egger	20	0.0576	-0.2223~0.3375	0.6914			-0.0052	0.4107	
	Weighted median	20	-0.0465	-0.2118~0.1189	0.5818					
	MR.RAPS	20	-0.0585	-0.1856~0.0687	0.3674					
	MR-PRESSO	20	-0.0517	-0.1676~0.0642	0.3931					0.3001
Gln	IVW	25	0.0134	-0.0543~0.0810	0.6988	23.0452	0.5171			
	MR-Egger	25	-0.0147	-0.1069~0.0775	0.7574			0.0027	0.3890	
	Weighted median	25	0.0084	-0.0749~0.0917	0.8434					
	MR.RAPS	25	0.0107	-0.0592~0.0806	0.7641					
	MR-PRESSO	25	0.0134	-0.0530~0.0797	0.6964					0.5956
His	IVW	8	-0.0944	-0.2569~0.0681	0.2548	3.7915	0.8035			
	MR-Egger	8	0.1204	-0.6743~0.9151	0.7765			-0.0145	0.6078	
	Weighted median	8	-0.0442	-0.2510~0.1627	0.6757					
	MR.RAPS	8	-0.0950	-0.2630~0.0731	0.2680					
	MR-PRESSO	8	-0.0944	-0.2140~0.0252	0.1657					0.8308
Ile	IVW	7	0.1744	0.0082~0.3405	*0.0397*†	6.4597	0.3737			
	MR-Egger	7	0.3534	-0.1097~0.8165	0.1950			-0.0095	0.4520	
	Weighted median	7	0.1818	-0.0308~0.3943	0.0937					
	MR.RAPS	7	0.1795	0.0086~0.3505	*0.0396*					
	MR-PRESSO	7	0.1744	0.0082~0.3405	0.0854					0.3640
Leu	IVW	9	0.1255	-0.0320~0.2831	0.1183	9.7951	0.2797			
	MR-Egger	9	-0.0909	-0.4174~0.2357	0.6025			0.0136	0.1887	
	Weighted median	9	0.0762	-0.0876~0.2400	0.3618					
	MR.RAPS	9	0.1206	-0.0559~0.2972	0.1804					
	MR-PRESSO	9	0.1255	-0.0320~0.2831	0.1569					0.3687
Phe	IVW	7	-0.1068	-0.2243~0.0108	0.0750	2.5878	0.8585			
	MR-Egger	7	-0.1174	-0.3757~0.1409	0.4139			0.0008	0.9315	
	Weighted median	7	-0.1132	-0.2502~0.0238	0.1053					
	MR.RAPS	7	-0.1070	-0.2281~0.0141	0.0832					
	MR-PRESSO	7	-0.1068	-0.184~-0.0296	*0.0351*					0.9067
Tyr	IVW	24	-0.0063	-0.0914~0.0787	0.8837	24.2904	0.3879			
	MR-Egger	24	-0.0047	-0.1315~0.1220	0.9422			-0.0001	0.9731	
	Weighted median	24	0.0414	-0.0715~0.1543	0.4721					
	MR.RAPS	24	-0.0006	-0.0903~0.0891	0.9898					
	MR-PRESSO	24	-0.0063	-0.0914~0.0787	0.8850					0.3875
Val	IVW	10	0.1042	-0.0167~0.2250	0.0911	4.6955	0.8600			
	MR-Egger	10	0.1185	-0.0996~0.3367	0.3180			-0.0011	0.8807	
	Weighted median	10	0.0920	-0.0496~0.2335	0.2027					
	MR.RAPS	10	0.1045	-0.0201~0.2292	0.1002					
	MR-PRESSO	10	0.1042	0.0169~0.1914	*0.0441*					0.8787

AAs, amino acids; Ala, alanine; Gln, glutamine; His, histidine; Ile, isoleucine; Leu, leucine; Phe, phenylalanine; Tyr, tyrosine; Val, valine; BMD, bone mineral density; LS-BMD, lumbar spine BMD; SNPs, single nucleotide polymorphisms; MR, Mendelian randomization; IVW, inverse variance weighted; MR.RAPS, MR Robust Adjusted Profile Score; MR-PRESSO, MR Pleiotropy RESidual Sum and Outlier; Effect, the causal effects of 1-SD increase of AAs on BMD; SD, standard deviation; CI, confidence interval.

^†^the italic MR p-value was considered nominally significant at p＜0.05.

**Table 3:** MR estimates of the causal effects of AAs on FA-BMD using various analysis methods.

Exposures	Methods	Number of SNPs	Effect	95% CI	MR p-value	Cochran Q statistic	Heterogeneity p-value	MR-Egger		MR-PRESSO
								Intercept	Intercept p-value	Global test p-value
Ala	IVW	24	0.0389	-0.1381~0.2159	0.6668	19.2259	0.6880			
	MR-Egger	24	0.0722	-0.3677~0.5122	0.7506			-0.0015	0.8726	
	Weighted median	24	0.0222	-0.2239~0.2683	0.8599					
	MR.RAPS	24	0.0466	-0.1374~0.2305	0.6198					
	MR-PRESSO	24	0.0389	-0.1230~0.2007	0.6421					0.7131
Gln	IVW	31	-0.0081	-0.1267~0.1106	0.8941	35.6282	0.2205			
	MR-Egger	31	-0.0385	-0.2100~0.1330	0.6632			0.0027	0.6294	
	Weighted median	31	-0.0068	-0.1519~0.1383	0.9266					
	MR.RAPS	31	-0.0129	-0.1401~0.1143	0.8425					
	MR-PRESSO	31	-0.0081	-0.1267~0.1106	0.8950					0.2835
His	IVW	9	0.1224	-0.1562~0.4011	0.3890	6.5817	0.5824			
	MR-Egger	9	0.0813	-0.7800~0.9425	0.8585			0.0026	0.9239	
	Weighted median	9	0.0325	-0.3272~0.3923	0.8594					
	MR.RAPS	9	0.1123	-0.1770~0.4016	0.4467					
	MR-PRESSO	9	0.1224	-0.1303~0.3752	0.3701					0.5349
Ile	IVW	7	0.2292	-0.0542~0.5126	0.1129	3.4614	0.7491			
	MR-Egger	7	0.1861	-0.5722~0.9443	0.6508			0.0023	0.9089	
	Weighted median	7	0.1504	-0.1960~0.4968	0.3949					
	MR.RAPS	7	0.2304	-0.0628~0.5235	0.1235					
	MR-PRESSO	7	0.2292	0.0140~0.4445	0.0819					0.7540
Leu	IVW	9	0.1661	-0.0865~0.4187	0.1976	5.9488	0.6530			
	MR-Egger	9	0.0499	-0.5036~0.6033	0.8648			0.0073	0.6577	
	Weighted median	9	0.0755	-0.2373~0.3884	0.6361					
	MR.RAPS	9	0.1608	-0.1005~0.4222	0.2277					
	MR-PRESSO	9	0.1661	-0.0518~0.3839	0.1735					0.6342
Phe	IVW	7	-0.2717	-0.4816~-0.0619	***0.0111*** *	4.1909	0.6509			
	MR-Egger	7	-0.1617	-0.6234~0.3000	0.5230			-0.0080	0.6224	
	Weighted median	7	-0.2880	-0.5314~-0.0446	*0.0204* ^†^					
	MR.RAPS	7	-0.2726	-0.4893~-0.0560	*0.0137*					
	MR-PRESSO	7	-0.2717	-0.4471~-0.0964	*0.0229*					0.7533
Tyr	IVW	26	-0.0384	-0.1897~0.1130	0.6192	27.9032	0.3123			
	MR-Egger	26	0.0401	-0.1850~0.2652	0.7303			-0.0063	0.3643	
	Weighted median	26	-0.0729	-0.2789~0.1330	0.4876					
	MR.RAPS	26	-0.0626	-0.2117~0.0865	0.4107					
	MR-PRESSO	26	-0.0384	-0.1897~0.1130	0.6236					0.2910
Val	IVW	13	0.0429	-0.1769~0.2627	0.7018	15.4376	0.2184			
	MR-Egger	13	0.2316	-0.1739~0.637	0.2868			-0.0124	0.3024	
	Weighted median	13	0.0880	-0.1717~0.3477	0.5065					
	MR.RAPS	13	0.0991	-0.1241~0.3224	0.3840					
	MR-PRESSO	13	0.0429	-0.1769~0.2627	0.7085					0.2297

AAs, amino acids; Ala, alanine; Gln, glutamine; His, histidine; Ile, isoleucine; Leu, leucine; Phe, phenylalanine; Tyr, tyrosine; Val, valine; BMD, bone mineral density; FA-BMD, forearm BMD; SNPs, single nucleotide polymorphisms; MR, Mendelian randomization; IVW, inverse variance weighted; MR.RAPS, MR Robust Adjusted Profile Score; MR-PRESSO, MR Pleiotropy RESidual Sum and Outlier; Effect, the causal effects of 1-SD increase of AAs on BMD; SD, standard deviation; CI, confidence interval.

*the bold and italic MR p-value was considered statistically significant at a Bonferroni-corrected p＜0.0125.

^†^the italic MR p-value was considered nominally significant at p＜0.05.

**Table 4:** MR estimates of the causal effects of AAs on TB-BMD using various analysis methods.

Exposures	Methods	Number of SNPs	Effect	95% CI	MR p-value	Cochran Q statistic	Heterogeneity p-value	MR-Egger		MR-PRESSO
								Intercept	Intercept p-value	Global test p-value
Ala	IVW	27	-0.0227	-0.0961~0.0507	0.5441	31.4677	0.2113			
	MR-Egger	27	-0.1454	-0.3275~0.0366	0.1300			0.0055	0.1628	
	Weighted median	27	0.0254	-0.0741~0.1249	0.6173					
	MR.RAPS	27	-0.0251	-0.1033~0.0531	0.5292					
	MR-PRESSO	27	-0.0227	-0.0961~0.0507	0.5494					0.2137
Gln	IVW	29	-0.0168	-0.0622~0.0285	0.4669	30.0697	0.3599			
	MR-Egger	29	-0.0469	-0.1095~0.0157	0.1536			0.0026	0.1898	
	Weighted median	29	-0.0230	-0.0774~0.0313	0.4062					
	MR.RAPS	29	-0.0165	-0.0641~0.0311	0.4960					
	MR-PRESSO	29	-0.0168	-0.0622~0.0285	0.4729					0.3548
His	IVW	10	0.0659	-0.0724~0.2043	0.3501	16.4419	0.0582			
	MR-Egger	10	0.3130	-0.1051~0.7311	0.1804			-0.0151	0.2560	
	Weighted median	10	0.1106	-0.0209~0.2422	0.0992					
	MR.RAPS	10	0.0973	-0.0140~0.2085	0.0866					
	MR-PRESSO	10	0.0659	-0.0724~0.2043	0.3745					0.0786
Ile	IVW	8	0.1601	0.0604~0.2597	***0.0016*** *	3.6813	0.8157			
	MR-Egger	8	0.2386	-0.0433~0.5204	0.1482			-0.0041	0.5808	
	Weighted median	8	0.1442	0.0207~0.2677	*0.0221*†					
	MR.RAPS	8	0.1608	0.0573~0.2643	***0.0023***					
	MR-PRESSO	8	0.1601	0.0878~0.2323	***0.0034***					0.8277
Leu	IVW	12	0.0759	-0.0104~0.1622	0.0847	11.2878	0.4195			
	MR-Egger	12	0.0693	-0.1446~0.2832	0.5398			0.0004	0.9482	
	Weighted median	12	0.1117	-0.0002~0.2237	0.0505					
	MR.RAPS	12	0.0925	0.0041~0.1808	*0.0402*					
	MR-PRESSO	12	0.0759	-0.0104~0.1622	0.1126					0.4493
Phe	IVW	8	-0.0406	-0.1168~0.0355	0.2956	1.9472	0.9627			
	MR-Egger	8	-0.0667	-0.2198~0.0864	0.4259			0.0018	0.7139	
	Weighted median	8	-0.0629	-0.1571~0.0313	0.1906					
	MR.RAPS	8	-0.0407	-0.1190~0.0376	0.3084					
	MR-PRESSO	8	-0.0406	-0.0808~-0.0005	0.0878					0.9229
Tyr	IVW	20	-0.1091	-0.1863~-0.0320	***0.0055***	20.5048	0.3648			
	MR-Egger	20	-0.0037	-0.1268~0.1194	0.9536			-0.0066	0.0596	
	Weighted median	20	-0.0477	-0.1623~0.0669	0.4147					
	MR.RAPS	20	-0.1142	-0.1932~-0.0351	***0.0046***					
	MR-PRESSO	20	-0.1091	-0.1863~-0.0320	*0.0121*					0.3302
Val	IVW	15	0.0953	0.0251~0.1655	***0.0078***	10.0451	0.7589			
	MR-Egger	15	0.0820	-0.0534~0.2174	0.2563			0.0010	0.8260	
	Weighted median	15	0.0918	0.0007~0.1829	*0.0484*					
	MR.RAPS	15	0.0979	0.0254~0.1705	***0.0082***					
	MR-PRESSO	15	0.0953	0.0358~0.1548	***0.0072***					0.8128

AAs, amino acids; Ala, alanine; Gln, glutamine; His, histidine; Ile, isoleucine; Leu, leucine; Phe, phenylalanine; Tyr, tyrosine; Val, valine; BMD, bone mineral density; TB-BMD, total body BMD; SNPs, single nucleotide polymorphisms; MR, Mendelian randomization; IVW, inverse variance weighted; MR.RAPS, MR Robust Adjusted Profile Score; MR-PRESSO, MR Pleiotropy RESidual Sum and Outlier; Effect, the causal effects of 1-SD increase of AAs on BMD; SD, standard deviation; CI, confidence interval.

*the bold and italic MR p-value was considered statistically significant at a Bonferroni-corrected p＜0.0125.

^†^the italic MR p-value was considered nominally significant at p＜0.05.

**Figure 1:**
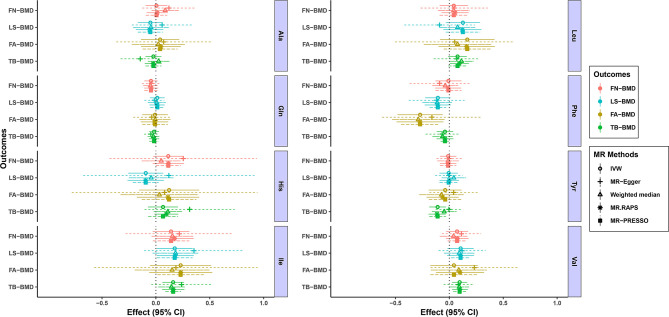
MR estimates of the associations between eight AA levels and BMD. The x-axis is the effects of AAs on BMD values. The vertical dashed line is the reference at effect = 0. The y-axis presents different BMD types, which are highlighted in different colors. Different MR methods are displayed with different line types. AAs, amino acids; BMD, bone mineral density; CI, confidence interval; IVW, inverse variance weighted; MR.RAPS, MR Robust Adjusted Profile Score; MR-PRESSO, MR Pleiotropy RESidual Sum and Outlier; Ala, alanine; Gln, glutamine; His, histidine; Ile, isoleucine; Leu, leucine; Phe, phenylalanine; Tyr, tyrosine; Val, valine; FN-BMD, femoral neck BMD; LS-BMD, lumbar spine BMD; FA-BMD, forearm BMD; TB-BMD, total body BMD.

**Figure 1** and **Tables 1**–**4** display the causal effects of AAs on BMD based on IVW, MR-Egger, Weighted median, MR.RAPS and MR-PRESSO methods. At the Bonferroni-corrected p threshold of 0.0125, the results provided evidence that genetically increased Ile (e.g., IVW: effect = 0.1601, 95% confidence interval [CI] = 0.0604 ~ 0.2597, p = 0.0016) and Val levels (e.g., IVW: effect = 0.0953, 95% CI = 0.0251 ~ 0.1655, p = 0.0078) were positively associated with TB-BMD (**Table 4**). The results revealed that genetically increased Tyr levels were negatively associated with TB-BMD (e.g., IVW: effect = -0.1091, 95% CI = -0.1863 ~ -0.0320, p = 0.0055) (**Table 4**). We did not observe the statistically significant associations between AAs and site-specific BMD (FN, LS and FA-BMD) (**Tables 1**–**3**), although Phe levels were negatively associated with FA-BMD at a nominal threshold (p<0.050) (e.g., IVW: effect = -0.2717, 95% CI=-0.4816 ~ -0.0619, p = 0.0111) (**Table 3**). For the other AAs, such as Ala and His, significant causal effects were not observed on FN, LS, FA and TB-BMD based on IVW, MR-Egger, Weighted median, MR.RAPS and MR-PRESSO methods (**Tables 1**–**4**).

We conducted heterogeneity analyses using the IVW method and conducted the pleiotropy analyses using the MR-Egger intercept test (**Tables 1**–**4**). Heterogeneity was not observed in the MR analyses for the causal associations of AAs on BMD changes (e.g, for the causal association between Ile and the TB-BMD, Q = 3.6813, p = 0.8157) (**Table 4**). Based on the MR-Egger intercept test, we did not find evidence of directional pleiotropy between the AA levels and BMD (e.g., Ile: intercept = -0.0041, p = 0.7601 for FN-BMD; intercept = -0.0095, p = 0.4520 for LS-BMD; intercept = 0.0023, p = 0.9089 for FA-BMD; and intercept = -0.0041, p = 0.5808 for TB-BMD) (**Tables 1**–**4**). The MR-PRESSO global test also revealed that no horizontal pleiotropic outliers were identified in the MR analyses (e.g., Ile: p = 0.1655 for FN-BMD; p = 0.3640 for LS-BMD; p = 0.7540 for FA-BMD; and p = 0.8277 for TB-BMD) (**Tables 1**–**4**). The results of MVMR adjusted for alcohol consumption, BMI and educational attainment were similar to the univariable MR results, with significant direct associations identified between Ile, Tyr, Val and TB-BMD (e.g., Ile: p = 0.0001 adjusted for alcohol consumption; p = 0.0097 adjusted for BMI; and p = 0.0058 adjusted for educational attainment) (**Tables S10–12**). The sample sizes of BMD traits in the current analysis are presented in **Table S13**. We calculated the proportions of AA variation explained by IVs ranging from 0.0056 to 0.0394. Under the current sample size and exposure variations, we provided the minimum and maximum detectable causal effects required to achieve 80% statistical power for the MR analysis, and they were located in the CI of our results. Therefore, our study had 80% power to detect a causal effect of 0.1535 g/cm^2 increase in TB-BMD per 1-SD increase of Ile levels, 0.0967 g/cm^2 increase in TB-BMD per 1-SD increase of Val levels and 0.1003 g/cm^2 decrease in TB-BMD per 1-SD increase of Tyr levels.

## Discussion

Molecular mechanism analyses have suggested that a number of AAs may be associated with BMD. Bone marrow stromal cells were demonstrated to express both intracellular and extracellular nutrient-sensing pathways for AAs, and certain AAs were described as potent stimulators of an increase in intracellular calcium, suggesting that AAs were important signaling molecules for normal cell function (36). Osteoblasts can express specialized AA receptors and transporters that enable the adjustment of cellular bioenergetics according to fluctuations in AA availability (37). Some AA was a potent stimulus of growth hormone secretion, which in turn results in an increase in circulating levels of insulin-like growth factor-1 (IGF-1), a known anabolic stimulus for osteoblasts. Bone mass can be elevated by AA-induced increases in calcium absorption efficiency, osteoblast proliferation and bone mineralization, synthesis of type I collagen, circulating levels of IGF-1, reduced bone resorption, osteoclast attachment and suppressed osteoclast differentiation (38). AAs can also enhance intestinal calcium absorption *in vivo*, increase the secretion of Alk Phos and decrease the production of interleukin-6 from osteoblasts *in vitro*. Increases in Alk Phos and decreases in interleukin-6 levels may result in increases in bone collagen synthesis and bone formation and reduced bone resorption (38).

In the present study, we reported for the first time the causal associations between AAs and BMD through a MR analysis. We provided evidence to support the causal effects of Ile,Val and Tyr on TB-BMD. In the MR study, we used strong IVs from the summary statistics of the largest GWAS conducted for AAs and BMD. We employed a range of methods known to control for pleiotropy and checked the heterogeneity, and we obtained highly consistent results. Pleiotropic effects were detected by using the MR-Egger intercept and MR-PRESSO method. Using the MR design, we could mitigate the confounding factors due to the application of Mendel’s second law of the random assortment of alleles. Reverse causality was also prevented because genetic variants were fixed at conception and could not be affected by disease processes. The results above showed that the presence of pleiotropic SNPs was minimal. Besides the univariable MR analysis, we also conducted the MVMR analysis taking into account the effect of some potential risk factors of BMD. We found the results were stable after adjusting for these risk factors. In addition, we also calculated the power of MR. Taken together, our MR results have high precision and stability to support the evidence.

Val, Leu, and Ile are branched-chain AAs (BCAAs) that are critical for the maintenance of bone strength and density and associated with greater muscle and fat mass (39). BCAAs have a direct effect on the initiation of mRNA translation and are the most potent stimulator of muscle protein synthesis, which is critical for the maintenance of adequate bone strength and density (40). In our study, we found that Ile and Val were positively associated with TB-BMD. Tyr, Phe and His are aromatic amino acids (AAAs) involved in protein synthesis. AAAs and their metabolites are involved in the synthesis of various secondary metabolites, including pigment compounds, plant hormones and biological polymers (41). The molecular mechanisms underlying the associations between AAAs and bone metabolism have been partially revealed. AAAs reduced the expression of the calcitonin receptor, carbonic anhydrase II and cathepsin K in osteoclasts *in vitro*, which may suppress osteoclast differentiation (42). Increasing the intake of AAAs might stimulate an increase in the circulating levels of IGF-1 and influence calcium homeostasis, which is involved in the stimulation of mature osteoblasts and regulates skeletal growth (43, 44). However, Le et al. (45) suggested that dietary AAA intake was not significantly associated with hip fractures, hip BMD, or any measurements of body composition. Our study support the negative causal effects of Tyr and Phe on BMD, although the findings indicated that Phe was negatively associated with FA-BMD at a nominal threshold. The negative associations between AAs and BMD were surprising, although some AAs were reported to cause bone loss and increase the risk of fracture. Higher homocysteine (Hcy) was associated with significant BMD decline and independently associated with a higher risk of fracture (8). The MR results from Wang et al. (46) also revealed a negative association between Hcy and BMD. However, they also indicated that decreased plasma Hcy was not associated with FN-BMD, LS-BMD and the risk for bone fracture. *In vitro* studies have revealed that Hcy might also promote collagen accumulation in bone, contribute to decreased bone strength and reduce bone blood flow (47), thus suggesting a pathogenic role of Hcy in bone health. A cross-sectional study involving a total of 773 Taiwanese women revealed that elevated Gln was significantly associated with low BMD (48). Gln might convert to glutamate, which would lead to bone resorption through the expression of glutamate receptors on bone cells, especially osteoclasts. This finding explained the association between elevated Gln and low BMD (48). However, we did not identify causal associations between Gln and BMD. Currently, only one clinical study has been published about the association between Ala and BMD. A cross-sectional study (49) of 103 patients with spinal cord injury found that higher alanine levels were not related to BMD after controlling for confounders, including demographic and injury-related characteristics and calcium intake. Our results did not support the causal associations between Ala and BMD. Although the causal associations were found between AAs and TB-BMD, we still did not support the causal associations between AAs and site-specific BMD (FN, LS and FA-BMD). This result also suggested that the effects of circulating AAs on bone metabolism might be systemic rather than local. The molecular mechanism of function of AAs on the bone metabolism also supported the hypothesis (36–38).

Although the design of MR analyses makes this method less susceptible to confounders than other observational studies, limitations still exist. First, we only evaluated the association between a single AA and BMD and did not consider the interactions between the AAs and the interactions with other nutritional factors, such as calcium, which might lead to potential pleiotropy. This limitation might cause the inconclusive causal associations between serum AA levels and BMD. However, we assessed potential pleiotropy using the MR-Egger method and MR-PRESSO method. We also used the PhenoScanner tool to exclude the SNPs associated with confounders. Hence, although the risk of a residual horizontal pleiotropic effect cannot be ruled out, it likely did not change the conclusions of this study in a clinically meaningful way. Second, we did not perform age and gender stratification for the population, which are two essential factors that can affect BMD (1). However, excluding these processes likely did not have a large effect on our analyses because of the large sample size included for AAs and BMD, which might have reduced the bias. Second, most of the population in the original GWAS were from European ancestry, but the participants in the TB-BMD GWAS were of mixed ancestry. The population stratification may not have been completely ruled out and may have influenced the causal estimates, although most participants were from population-based cohorts of European ancestry in the TB-BMD GWAS (22). Last, we did not thoroughly explore the mechanism underlying the causality between AAs and BMD. Therefore, mechanistic research should focus on specific AAs at cellular and individual levels in the future.

## Conclusion

In summary, we provided precise evidence that the levels of certain AAs in the serum, namely, Ile and Val, were positively associated with TB-BMD; and Tyr was negatively associated with TB-BMD. We did not observe the statistically significant associations between AAs and site-specific BMD (FN, LS and FA-BMD). These findings underscore the important role that serum AAs play in the development of osteoporosis and provide evidence that osteoporosis can be treated and prevented by supplementing certain AAs. Future studies are needed to investigate the potential mechanisms by which AAs influence bone metabolism and to examine the potential role of these mechanisms in the treatment of osteoporosis.

## Data Availability Statement

The original contributions presented in the study are included in the article/**Supplementary Material**. Further inquiries can be directed to the corresponding author.

## Author Contributions

YT, JH, and ZC conceptualized and designed the study. ZC and BH provided the package codes in R language and analyzed the data in the study. ZC drafted the manuscript. HF gave constructive suggestions when writing the manuscript. All authors contributed to the article and approved the submitted version.

## Funding

This work was supported by grants from National Natural Science Foundation of China (Grant No.51873214).

## Conflict of Interest

The authors declare that the research was conducted in the absence of any commercial or financial relationships that could be construed as a potential conflict of interest.

## Publisher’s Note

All claims expressed in this article are solely those of the authors and do not necessarily represent those of their affiliated organizations, or those of the publisher, the editors and the reviewers. Any product that may be evaluated in this article, or claim that may be made by its manufacturer, is not guaranteed or endorsed by the publisher.
